# Video-telecare collaborative pain management during COVID-19: a single-arm feasibility study

**DOI:** 10.1186/s12875-023-02052-2

**Published:** 2023-06-29

**Authors:** Daniel G. Rogers, Joseph W. Frank, Danielle M. Wesolowicz, Charlotte Nolan, Allison Schroeder, Caroline Falker, Audrey Abelleira, Brent A. Moore, William C. Becker, Sara N. Edmond

**Affiliations:** 1 VA Connecticut Healthcare System, West Haven, CT USA; 2 Department of Psychiatry, Yale School of Medicine, West Haven, USA; 3 VA Eastern Colorado Health Care System, Aurora, USA; 4 University of Colorado School of Medicine, Aurora, USA; 5 Department of Internal Medicine, Yale School of Medicine, New Haven, USA

**Keywords:** Chronic pain, Long-term opioid therapy, Buprenorphine, Telemedicine

## Abstract

**Background:**

Chronic pain is among the most common conditions presenting to primary care and guideline-based care faces several challenges. A novel pain management program, Video-Telecare Collaborative Pain Management (VCPM), was established to support primary care providers and meet new challenges to care presented by the COVID-19 pandemic.

**Methods:**

The present single-arm feasibility study aimed to evaluate the feasibility and acceptability of VCPM and its components among U.S. veterans on long-term opioid therapy for chronic pain at ≥ 50 mg morphine equivalent daily dose (MEDD). VCPM consists of evidence-based interventions, including opioid reassessment and tapering, rotation to buprenorphine and monitoring, and encouraging behavioral pain and opioid-use disorder self-management.

**Results:**

Of the 133 patients outreached for VPCM, 44 completed an initial intake (33%) and 19 attended multiple VCPM appointments (14%). Patients were generally satisfied with VCPM, virtual modalities, and provider interactions. Nearly all patients who attended multiple appointments maintained a buprenorphine switch or tapered opioids (16/19; 84%), and buprenorphine switches were generally reported as acceptable by patients. Patients completing an initial intake with VCPM had reduced morphine equivalent daily dose after three months (means = 109 mg MEDD vs 78 mg), with greater reductions among those who attended multiple appointments compared to intake only (Δ_MEDD_ = -58.1 vs. -8.40). Finally, 29 referrals were placed for evidence-based non-pharmacologic interventions.

**Conclusion:**

Pre-defined feasibility and acceptability targets for VCPM and its components were broadly met, and preliminary data are encouraging. Novel strategies to improve enrollment and engagement and future directions are discussed.

## Introduction

Chronic pain is among the most commonand debilitating conditions in the U.S. and worldwide [1, 2], and disproportionately affects U.S. Veterans compared to the general U.S. population [1, 3, 4]. Historically, chronic pain management relied heavily on long-term opioid therapy (LTOT), though emerging evidence suggests uncertain or low efficacy [5–7], in addition to adverse events including opioid use disorder (OUD) and opioid overdose [8]. As such, clinical practice guidelines recommend non-opioid pharmacologic and non-pharmacologic pain management approaches, continual reassessment of the benefits and harms of continued LTOT, and consideration of tapering or discontinuing LTOT if harms outweigh benefits or rotation to buprenorphine if clinically indicated, though implementation of these strategies in primary care settings face many barriers.

The COVID-19 pandemic disrupted in-person healthcare, including access to many non-pharmacologic pain management approaches. This led to an urgent need for adaptation of clinical care, particularly for patients with chronic pain prescribed LTOT whose access to evidence-based pain care may be limited due to public health precautions. To address primary care and COVID-19 related challenges, we aimed to evaluate the feasibility and acceptability of a program called Video-Telecare Collaborative Pain Management (VCPM) in preparation for a larger-scale trial. VCPM comprises three evidence-based components: opioid reassessment and tapering, buprenorphine switch and monitoring, and behavioral pain and OUD self-management [9–16].

## Methods

### Design and patients

This single-arm feasibility study was funded as a Veterans Health Administration (VHA) Health Services Research & Development Rapid Response Project (C19 20–397; Oct. 2020—July 2021), a rapid funding initiative to respond to challenges related to the COVID-19 pandemic. The study took place in two VHA healthcare systems located in the northeast and mountain regions and was deemed quality improvement by the two participating VHA Institutional Review Boards.

Eligible patients were Veterans on LTOT for chronic pain at ≥ 50 mg morphine equivalent daily dose (MEDD). Exclusion criteria included a diagnosis of dementia or moderate-severe cognitive impairment, unstable or severe untreated psychiatric disorder or medical disease that required hospitalization, documentation of suspected controlled substance diversion, or inability to communicate by phone. Eligible patients were identified through referrals from primary care providers to the participating VHA site’s pain management teams (PMTs) or through direct patient outreach using internal opioid risk reports to identify potentially eligible patients.

### Procedure

VCPM is a multicomponent intervention led by pain-trained clinical pharmacy practitioners (CPPs) and supported by a collaborating physician, modeled after the collaborative care intervention in the SPACE trial [17]. The physicians and pharmacists are trained in opioid reassessment and taper, buprenorphine switch and monitoring, and behavioral self-management strategies. Each participating healthcare system was staffed by one CPP and one collaborating physician. A semi-structured interview guide was provided alongside standardized note templates to ensure fidelity of the intervention among the two teams.

Eligible patients received an information packet via mail or email and were then contacted by study staff to schedule an intake evaluation with the CPP. In collaboration with the supporting physician, a pain management plan was created and presented to the patient (e.g., opioid taper, buprenorphine switch). For data collection purposes, duration of active intervention was set for 90 days with follow-ups increasingly spaced-out over time, though duration of intervention and frequency of follow-ups were ultimately determined on a case-by-case basis according to patient’s clinical situation (e.g., some patients were discharged prior to 90 days of goals were met; other patients continued working with CPPs beyond 90 days). Patient preference was incorporated into virtual treatment modality (video vs. telephone) and information receipt (web, email, mail). If a patient declined follow-up, VCPM recommendations were sent to their primary care provider. Patients also completed outcome measurements three months after their baseline visit.

### Measures and data sources

#### Feasibility and acceptability

The primary outcomes of interest were feasibility and acceptability of VCPM, measured by 1) number of referrals through participating PMTs or direct outreach, 2) enrollment as determined by completion of a VCPM intake, 3) continued engagement with the VCPM program (i.e., attended one or more follow-up appointments after intake), and 4) patient-reported acceptability. Feasibility and acceptability of opioid reassessment and taper or buprenorphine switch were based on the total number of patients who engaged in an opioid taper or buprenorphine switch. Acceptability of VCPM was assessed using an 18-item questionnaire assessing the impact of COVID-19, healthcare-related use of technology, and questions regarding satisfaction with virtual visits, the VCPM pain management model, and buprenorphine (if trialed). Questions included willingness to engage with VCPM or to trial buprenorphine, success of interventions, and confidence in recommending the intervention (11-point scales from 0 = not at all to 10 = very). We used electronic health record data and follow-up phone evaluations to collect additional data.

#### Electronic health record data

We obtained data through chart review of electronic health records and review of provider documentation. Variables of interest included demographic data, VHA service connection, pain generator, medical and mental health comorbidities (aggregated into risk index for overdose or serious opioid‐induced respiratory depression (RIOSORD) comorbidities [18], substance use disorders, and mental health disorders). Provider documentation and administrative data was used to confirm engagement with VCPM, including opioid taper, buprenorphine switch and maintenance, MEDD after three months, and engagement with non-pharmacologic pain treatments (e.g., interventional pain, complementary and integrative health).

#### PEG-3

The PEG-3 is a reliable and valid three-item measure of pain intensity, interference with enjoyment of life, and interference with general activity [19]. Items are rated on an 11-point Likert-like scale (0 = no pain/does not interfere to 10 = worst pain/completely interferes) and be interpreted individually or as a mean.

#### Questionnaire on the Quality of Patient-Physician Interaction (QQPPI)

This study utilized a modified version of the 14-item QQPPI [20], a measure designed to quantify patient satisfaction with prover interactions. Each question was modified to replace “doctor” or “physician” with the more inclusive term, “clinician.” One question was omitted due to the virtual format (“the physician gave me a thorough examination”) and two pain-specific questions were added (“the clinician talked to me about pain” and “the clinician talked to me about options to manage my pain other than medications”). The modified version of the QQPPI included 15 questions rated on a 5-point scale (1 = I do not agree to 5 = I fully agree) that are summed, for a score ranging from 15 to 75, with higher scores reflecting greater satisfaction.

## Statistical analysis

Feasibility and acceptability outcomes were evaluated based on descriptive statistics with defined a priori criteria, used to determine whether further evaluation was warranted. Poor feasibility or acceptability were defined as follows: 1) the majority of patients declining to trial a buprenorphine switch (> 50%), 2) low treatment satisfaction (> 50% negative satisfaction), or 3) low engagement in follow-up (< 50% attending follow-up). Given the nature of this small pilot quality improvement project, secondary outcomes were evaluated using descriptive statistics only. Analyses were performed using SPSS for Windows (Version 28; SPSS, IBM Corp, Armonk, NY).

## Results

### Patient characteristics

VCPM patients were primarily male (42/44; 96%) and white (38/44; 86%), with a mean age of 63.5 years (SD = 8.5), and about half were from urban locations (55%) (see Table 1). The most common pain generators were low back pain (86%) and osteoarthritis (59%). The sample was medically complex, with 90% including a RIOSORD comorbidity, 50% a mental health diagnosis, and 25% a substance use disorder.

**Table 1:** Demographic Characteristics of Patients

VCPM patient characteristics	n (%) or mean (SD)
Gender, n (%)	
Male	42 (95.5)
Female	2 (4.5)
Age, mean (SD)	63.5 (8.5)
Rural, n (%)	24 (54.5%)
Race/Ethnicity, n (%)	
Black	3 (6.8)
White	38 (86.4)
Other	3 (6.8)
Ethnicity, n (%)	
Hispanic or Latino	11 (25.6)
Pain Generator, n (%)	
Back Pain	38 (86.3)
Fibromyalgia	2 (4.5)
Neck Pain	16 (36.3)
Osteoarthritis	26 (59.1)
Neuropathic Pain	15 (34.1)
Headache	9 (20.5)
Other MSK	25 (56.8)
Other Rheumatological	8 (18.2)
Comorbidities, n (%)	
RIOSORD Comorbidity	40 (90%)
Substance Use Disorder	11 (25%)
Mental Health Disorder	22 (50%)
Morphine equivalent daily dose, mean (SD)	117.3 (100.3)
Location, n (%)	
VA Connecticut Healthcare System	17 (38.6)
VA Eastern Colorado Health Care System	27 (61.4)

### Feasibility and acceptability of VCPM

We contacted 133 patients to offer VCPM. Of those, 44 completed an intake evaluation (33%) and 19 continued VCPM engagement past intake (14%). Approximately three-quarters (32/44) of patients who completed intake evaluations completed follow-up evaluations.

Patients reported high satisfaction with both video visits (mean = 4.3/5) and phone visits (mean = 4.0/5), high willingness to engage with VCPM (mean = 8.8 / 10; SD = 2.5) and confidence in recommending the program (mean = 7.0 / 10; SD = 3.5), with no difference in satisfaction between those completing only an intake evaluation (*n* = 16) compared to those who were seen for multiple appointments (*n* = 16). Patients who continued in VCPM beyond the intake evaluation rated VCPM as more successful (means = 6.9 vs 4.1) and reported higher satisfaction with provider interactions (means = 70.2 vs 64.1) compared to those who completed an intake evaluation only.

### Feasibility and acceptability of buprenorphine switch

Individuals who continued engagement in VCPM had high rates of trialing buprenorphine (11/19; 58%) and of either maintaining a buprenorphine switch (9/19; 47%) or tapering full-agonist opioids (7/19; 37%) after three months. Those who trialed buprenorphine and completed the three-month survey (*n* = 9) rated, on average, their willingness to try buprenorphine as 7.8/10, view of buprenorphine as successful as 6.5/10, and confidence in recommending buprenorphine as 6.5/10.

### Secondary outcomes

Individuals who completed the VCPM intake evaluation reduced their MEDD three months later (means = 109 mg MEDD at intake vs 78 mg MEDD three months later). Individuals who continued engaging with VCMP for multiple appointments, compared to those who attended only an intake evaluation, exhibited a larger MEDD reduction after three months (Δ_MEDD_ = -58.1 vs -8.40).

Among individuals completing the intake evaluation, 29 referrals were placed for evidence-based non-pharmacologic interventions. Most referrals (11/29; 38%) went to complementary and integrative health programs (e.g., acupuncture, massage, chiropractor), followed by physical therapy (9/29; 31%), behavioral interventions (e.g., cognitive-behavioral therapy for pain; 6/29; 21%), and interventional pain programs (4/29; 14%).

On the three-month survey, individuals who continued VCPM engagement compared to those completing only the initial evaluation reported a lower pain intensity (6.1 vs 7.1/10), pain interference with enjoyment of life (6.0 vs 8.3/10), and pain interference with general activity (5.9 vs 7.4/10). When describing how pain has changed over the last three months, 44% of those who continued engagement reported improvement while 25% reported worsened pain. Among those who completed an intake evaluation only, 19% reported improvement and 38% reported worsened pain over the last three months.

## Discussion

The current quality improvement project examined the feasibility and acceptance of a novel virtual care model of pain management utilizing well-established components including opioid reassessment and tapering, buprenorphine switch and monitoring, and behavioral pain and OUD self-management [9–16].

Broadly, the project met the identified a priori feasibility and acceptability criteria for the virtual delivery component. Across 17 months, 133 Veterans were identified as eligible for VCPM, resulting in 44 intake evaluations (44/113; 33%). Of those who completed an intake evaluation, 19 patients also received follow-up care from a VCPM CPP (19/44; 43%). The project succeeded in engaging rural Veterans who made up nearly 50% of the sample, notable in the context that, only 14% of patients with an assigned primary care provider among these two participating VHA systems are in rural areas [21, 22]. However, enrolling patients receiving LTOT to a pain management team with a clear emphasis on opioid tapering or transition can be difficult given numerous potential barriers including stigma, concerns regarding changing medications and withdrawal, and the relative unfamiliarity of buprenorphine compared to traditional opioids [23]. Novel recruitment strategies may help to bolster enrollment. For instance, peer specialists, or patients with lived experience, may be effective in encouraging other patients to trial new approaches, and VHA is expanding its use of peer specialists into primary care clinics and other new settings.

Among those enrolled, acceptability of VCPM was high, with robust satisfaction for both virtual care modalities (i.e., phone and video conferencing). Patients reported high willingness to engage with virtual care and confidence recommending the program. As would be expected for individuals choosing to engage with the program, those who attended multiple appointments with the CPP generally rated the program as more successful than those completing only an intake evaluation. Future efforts will focus on understanding reasons for lack of engagement.

Similarly, a priori criteria for success of the buprenorphine switch component of VCPM were largely met. Among those engaged in VPCM, over half trialed buprenorphine and nearly all of those maintained their buprenorphine regimen at three-month follow-up. From a harm-reduction standpoint, VCPM was successful in tapering opioids or transitioning to buprenorphine with a majority of patients. Acceptability of the buprenorphine switch was generally favorable, with patients reporting moderate or high willingness to trial buprenorphine, view of the buprenorphine switch as successful, and confidence recommending to a friend.

Preliminary findings are encouraging, as individuals who continued to engage with VCPM exhibited greater MEDD reductions and indications of improved pain outcomes compared to those completing only a baseline intake. It is important to note, however, the single-arm nature and emphasis as a quality improvement project preclude comparisons to traditional, in-person pain management interventions.

The present quality improvement project provides initial support for VCPM and the virtual delivery of multi-modal pain management interventions. Within the VHA system, virtual interventions offer the ability to markedly increase the reach of programs designed to assist primary care providers, particularly among regions where maintaining a specialized care team is difficult. Larger effectiveness trials are needed to 1) compare VCPM to other pain management interventions and 2) ensure non-inferiority of virtual interventions and 3) evaluate VCPM among more diverse populations (e.g., across demographic variables and pain conditions).

## Data Availability

The datasets used and/or analyzed during the current study are available, de-identified, from the corresponding author on reasonable request.

## Abbreviations

LTOT: Long-term Opioid Therapy
OUD: Opioid Use Disorder
VCPM: Video-Telecare Collaborative Pain Management
VHA: Veterans Health Administration
MEDD: Morphine Equivalent Daily Dose
PMT: Pain Management Team
CPP: Clinical Pharmacy Practitioners
RISORD: Risk Index for Overdose or Serious Opioid‐Induced Respiratory Depression
QQPPI: Questionnaire on the Quality of Patient-Physician Interaction
